# Multi-model comparison of the economic and energy implications for China and India in an international climate regime

**DOI:** 10.1007/s11027-014-9549-4

**Published:** 2014-02-28

**Authors:** Daniel J. A. Johansson, Paul L. Lucas, Matthias Weitzel, Erik O. Ahlgren, A. B Bazaz, Wenying Chen, Michel G. J. den Elzen, Joydeep Ghosh, Maria Grahn, Qiao-Mei Liang, Sonja Peterson, Basanta K. Pradhan, Bas J. van Ruijven, P. R. Shukla, Detlef P. van Vuuren, Yi-Ming Wei

**Affiliations:** 1 Department of Energy and Environment, Chalmers University of Technology, 412 96 Gothenburg, Sweden; 2 PBL Netherlands Environment Assessment Agency, PO Box 303, 3720 AH Bilthoven, The Netherlands; 3 Kiel Institute for the World Economy, Kiellinie 66, 24105 Kiel, Germany; 4 Public Systems Group, Wing 3, Indian Institute of Management Ahmedabad, Vastrapur, Ahmedabad, 380015 India; 5 Energy, Environment, and Economy (3E) Research Institute, Tsinghua University, Beijing, 100084 China; 6 Institute of Economic Growth (IEG), University of Delhi, North Campus, Delhi, 110007 India; 7 Center for Energy and Environmental Policy Research, Beijing Institute of Technology, Beijing, 100081 China; 8 National Center for Atmospheric Research (NCAR), PO Box 3000, Boulder, CO 80305 USA; 9 Department of Geosciences, Utrecht University, Heidelberglaan 2, 3584 CS Utrecht, The Netherlands

**Keywords:** Climate policy, China, India, Costs, Energy

## Abstract

This paper presents a modeling comparison on how stabilization of global climate change at about 2 °C above the pre-industrial level could affect economic and energy systems development in China and India. Seven General Equilibrium (CGE) and energy system models on either the global or national scale are soft-linked and harmonized with respect to population and economic assumptions. We simulate a climate regime, based on long-term convergence of per capita carbon dioxide (CO_2_) emissions, starting from the emission pledges presented in the Copenhagen Accord to the United Nations Framework Convention on Climate Change and allowing full emissions trading between countries. Under the climate regime, Indian emission allowances are allowed to grow more than the Chinese allowances, due to the per capita convergence rule and the higher population growth in India. Economic and energy implications not only differ among the two countries, but also across model types. Decreased energy intensity is the most important abatement approach in the CGE models, while decreased carbon intensity is most important in the energy system models. The reduction in carbon intensity is mostly achieved through deployment of carbon capture and storage, renewable energy sources and nuclear energy. The economic impacts are generally higher in China than in India, due to higher 2010–2050 cumulative abatement in China and the fact that India can offset more of its abatement cost though international emission trading.

## Introduction

In the Copenhagen Accord (UNFCCC 2009) and the Cancún Agreements (UNFCCC 2010) under the United Nations Framework Convention on Climate Change (UNFCCC), countries worldwide agreed on limiting global average temperature increase to maximum 2 °C above the pre-industrial level. In order to reach this political target of staying below 2 °C above the pre-industrial level with a probability of more than 50 % global greenhouse gas emissions need be to be cut about 35–55 % by 2050 compared to the emissions level in 1990 (Rogelj et al. 2011). By 2005, about 50 % of the anthropogenic greenhouse gases accumulated in the atmosphere can be attributed to developed countries (Höhne et al. 2011). However, the greater share of future emissions is expected to come from developing countries. In fact, emissions from developing countries alone will soon exceed the global emission trajectory for reaching the 2 °C target (Clarke et al. 2009; Metz et al. 2002; Blanford et al. 2009). This implies that, even though universal participation in a climate regime is not necessary in the short-run, participation of rapidly developing countries is essential.

The current size and expected growth of the Chinese and Indian population and economy imply that these countries will have an important role in shaping the dynamics of the future global energy system and the carbon dioxide (CO_2_) emissions from combustion of fossil fuels (IEA 2011). At the same time, however, per capita income levels in both China and India are still much lower than those of developed countries. For India, this also holds for per capita emissions, while China’s average per capita CO_2_ emissions have reached almost similar levels as those of the EU (Olivier et al. 2012). Already, China and India have pledged emission intensity targets (i.e., reduction in emissions per unit of GDP) as part of the Cancún Agreements.^1^ While the aggregated pledges for all countries are able to reduce global emissions compared to baseline development, after 2020 deeper cuts beyond these pledges are required in order to achieve the 2 °C target.

Deep global emission cuts will however come at economic costs. There are many proposals discussed in literature that address how the global emission space, compatible with the 2 °C target, could be shared between different countries or regions, and the related costs and macro-economic implications. These effort sharing or emission allocation proposals have different participation levels, timing of reductions, as well as stringency and type of commitments (See an overview of proposals in e.g. Bodansky 2004; Kameyama 2004; Philibert 2005; Gupta et al. 2007; Den Elzen and Höhne 2008). Furthermore, there is a broad literature on the economic impact of different proposals, and many papers discuss how China and India would be affected (see Van Ruijven et al. 2012b and references therein).

The overall objective of this study is to develop an integrated modelling framework that enables policy and scenario analyses on how China and India can be affected by international climate policies. Specifically, we apply the framework to analyze the consequences for China and India of a climate policy scenario aiming at achieving the 2 °C target, using an effort sharing approach that aims for long-term convergence of per capita emission levels and staged participation of developing countries. The analysis focuses on (1) the impact on their energy systems; and (2) the direct mitigation costs and welfare implications.

We analyze these issues in a multi-model comparison approach involving seven models in a single framework. The modeling framework harmonizes and soft-links national and global as well as CGE and energy system models. It aims to yield a more consistent global and national perspective compared to the existing literature. Most past assessments of climate policy impacts in India or China have either been carried out in national models only (e.g. Shukla and Chaturvedi 2012; Shukla 1996; Fisher-Vanden et al. 1997 for India; and ERI 2009 for China), or within global models (e.g. Edenhofer et al. 2010; Luderer et al. 2012; Van Vuuren et al. 2003). There is also broad literature on model comparison studies (e.g. Clarke et al. 2009; Edenhofer et al. 2010), including the Energy Modeling Forum (EMF) and—more closely related to the context of this paper—the Asian Model Exercise (AME) (Calvin et al. 2012). Compared to the AME we make use of a smaller number of models. However, we provide greater consistency by harmonizing and soft-linking the models.

## Modeling framework

### Description of the models

Central to the modeling framework is the climate policy model FAIR (Framework to Assess International Regimes for the differentiation of commitments). It is used to construct the long-term global greenhouse gas emission pathway consistent with the 2 °C target and to derive regional emission targets by applying specific effort sharing approaches. Furthermore, six energy economic models are used to determine changes to the energy system and national costs of climate policy. These models differ in two important dimensions: they are either global or national models and they are either energy system models or CGE models. While the global models can capture international linkages and feedbacks, the national models account better for country specific details and can therefore analyze the national impacts of international climate policies in more detail. While energy system models include technological details of energy production and consumption technologies, CGE models account for macro-economic feedbacks, changes in energy service demand and shifts in trade. Central features of the models are presented in Table 1. For specific assumptions of each model we refer to Sections 2.1.1 and 2.1.2 and papers covering the details of each model.

**Table 1:** The table shows an overview of the key characteristics of the seven models included in the applied model framework

	FAIR	TIMER	DART	CEEPA	China MARKAL	IEG-CGE	MARKAL-India
Institute	Netherlands Environmental Assessment Agency (PBL)	Netherlands Environmental Assessment Agency (PBL)	Kiel Institute for the World Economy (IfW)	Beijing Institute of Technology (BIT)	Tsinghua University (TU)	Institute of Economic Growth (IEG)	Indian Institute of Management-Ahmedabad (IIM-A)
Model class	Climate policy model	Recursive dynamic energy system model	Recursive dynamic computable general equilibrium model (CGE)	Recursive dynamic computable general equilibrium model (CGE)	Energy system model with perfect foresight	Recursive dynamic computable general equilibrium model (CGE)	Energy system model with perfect foresight
Global or national coverage	Global (26 regions)	Global (26 regions)	Global (13 regions)	China	China	India	India
Household groups	NA	10 (urban and rural quintiles)	1 representative agent per region	2 (urban and rural)	2 (urban and rural)	9	1
Sectors	NA	5	12	24	5 sectors; 32 sub-sectors	18	5 Sectors; 46 end-use sectors
Energy resources/technologies	NA	Coal, oil, natural gas, modern biofuels, traditional biofuels, nuclear, solar, wind and hydro power	Coal, natural gas, oil, bio-energy, wind, solar and hydro power	Coal, natural gas, oil, bio-energy, nuclear, wind, solar and hydro power	Coal, natural gas, oil, bio-energy, nuclear, wind, solar and hydro power	Coal, natural gas, oil, bio-energy, nuclear, wind/solar and hydro power	Coal, natural gas, oil, bio-energy, nuclear, solar, wind and hydro power
Technology dynamics	Based on MAC curves from TIMER and other models	Capital stocks, penetration rate constraints, and learning by doing	Capital stocks, learning by doing, and autonomous energy efficiency improvements	Capital stocks, and autonomous energy efficiency improvements	Capital stocks, and penetration rate constraints	Capital stocks, energy efficiency improvement, total factor productivity growth, and efficiency improvements	Capital stocks, penetration rate constraints, and energy infrastructure
CCS	NA	Yes	Yes	No	Yes	Yes	Yes
Substitutes to petroleum as transport fuel	NA	Electricity, modern biomass, hydrogen	Not explicitly modeled	Not explicitly modeled	Yes	Not explicitly modeled	Electricity, modern biomass, hydrogen
Demand side measures^a^	Included in MAC	End use efficiency and conservation measures	End use efficiency and conservation measures	End use efficiency and conservation measures	End use efficiency measures	End use efficiency and conservation measures	End use efficiency measures

*NA* not applicable

^a^End use efficiency refers to technological measures that can be used to increase energy efficiency, while conservation measures refer to measures representing changes in energy service demand, e.g. price responsive service demand

The FAIR model links long-term climate targets and global emission reduction objectives with regional emissions allowances and abatement costs (Den Elzen and Lucas 2005; Den Elzen et al. 2008). It includes the models FAIR–SiMCaP (Simple Model for Climate Policy assessment) (Den Elzen et al. 2007) and the MAGICC 6 (Model for the Assessment of Greenhouse Gas Induced Climate Change) climate model (Meinshausen et al. 2011) to construct long-term cost effective global greenhouse emission pathways, consistent with long-term climate targets. Furthermore, the model includes an emission allocation model that calculates regional emission allowances for a wide range of effort sharing and emission allocation proposals (see Den Elzen and Lucas 2005). Finally, the FAIR model also include a cost model that uses a least-cost approach involving regional Marginal Abatement Cost (MAC) curves to determine regional mitigation costs, allowing offsetting mechanisms such as international emission trading. The MAC curves consider abatement of all major emission sources, including abatement of energy- and industry-related greenhouse gases emissions (based on the TIMER (The IMAGE Energy Regional model) energy model (see Section 2.1.1)), land-use CO_2_ emissions (based on the IMAGE model (Bouwman et al. 2006)), and emissions of non-CO_2_ greenhouse gases (based on Lucas et al. (2007)). The MAC curves account for technology change, including technology inertia and removal of implementation barriers.

#### Energy systems models

Three energy-system models are used, which take into consideration the long-term dynamics of demand and supply of energy services based on large sets of existing and future technologies (that today are in demonstration phase) that can play a role in the future energy system. The technologies are linked together by energy (and/or material) flows. TIMER^2^ is a recursive dynamic global energy system model that describes the long-term dynamics of the production and consumption of energy for 26 world regions (Van Vuuren et al. 2006, 2007). China MARKAL (Chen 2005; Chen et al. 2007, 2010) and MARKAL-India (Shukla 1997; Shukla et al. 2008) are national energy system optimization models based on the MARKAL (MARKet ALlocation) modeling system (Fishbone and Abilock 1981).

All three models account for energy-related CO_2_ emissions from fossil fuels, while TIMER accounts for energy and industry related emissions of all greenhouse gases included in the Kyoto Protocol under the UNFCCC. The models include most primary energy resources and energy conversion technologies (and their costs and conversion efficiencies), including fossil fuels, biomass, nuclear power and several renewable energy resources/technologies (solar photovoltaics (PV), wind and hydro power). A carbon tax can be used to induce a response such as increased use of low or zero-carbon technologies, energy efficiency improvements and end-of-pipe emission reduction technologies such as carbon capture and storage (CCS).

In TIMER, model behavior is mainly determined by substitution processes of various technologies based on long-term fossil fuel prices and fuel preferences. These two factors drive multi-nominal logit models that describe investments in new energy production and consumption capacity. As capital is only replaced at the end of the technical lifetime demand for new capacity is limited. The long-term prices are determined by resource depletion (fossil and renewable energy resources) and technology development. Technology development is determined by endogenous learning curves and exogenous assumptions. The MARKAL models are dynamic linear programming energy system optimization models, encompassing extraction, transformation and end-use of energy. They are driven by a set of demands for energy services and their objective function is the long-term discounted energy system cost. Investment decisions are taken on the basis of least-cost optimization of the energy system, taking into account learning and depletion of resources. The optimizing feature ensures that the models compute a partial economic equilibrium of the energy system (Loulou et al. 1997).

#### Computable General Equilibrium (CGE) models

Three multi sectoral, recursive dynamic CGE models are used in this model exercise, describing the behavior of economic agents and their interactions in the macroeconomic system. DART (Dynamic Applied Regional Trade Model) is a global model calibrated to the data set of the Global Trade Analysis Project (Narayanan and Walmsley 2008) and aggregated to 13 regions (Klepper et al. 2003; Kretschmer et al. 2009). In addition, two single-country CGE models are applied: CEEPA (China Energy and Environmental Policy Analysis) describes the Chinese economy (Liang et al. 2007; Liang and Wei 2012), based on input-output data of the National Bureau of Statistics PR China (2009). IEG-CGE (Institute of Economic Growth-CGE) describes the Indian economy (Pradhan and Ghosh 2012a, b) and is based on a social accounting matrix (Ojha et al. 2009; Pradhan et al. 2006). The single country models capture characteristics of the labor and energy markets of the respective countries. For international trade, all models assume imperfect substitution between imported and domestically produced goods (Armington 1969).

DART uses one representative agent for each region that comprises private households and the government sector, and receives all income generated by providing sectorally mobile but regionally immobile primary factors (capital, labor, land and natural resources) to the production process. In CEEPA, consumers are divided into households, enterprises and government. Considering the current energy and emission intensive international trade structure of China, a foreign account was included. IEG-CGE divides consumers into nine household groups (based on socioeconomic characteristics), enterprises and government. DART and IEG-CGE model consumption as a linear expenditure system.

All models account for energy related CO_2_ emissions from combustion of fossil fuels. All models have introduced electricity generation technologies with low or zero CO_2_ emissions. In the DART model, making use of information provided by the TIMER model, the electricity sector was split into conventional generation and new generation technologies from four renewable energy sources; additionally gas and coal generation with CCS is introduced as a latent technology (Weitzel 2010). Different electricity generation technologies are assumed to be perfect substitutes, each technology has a convex cost function and exhibits learning-by-doing, i.e., the cost of the technology decreases as the cumulative use of it increases. In IEG-CGE and CEEPA, different electricity generation options are non-perfect substitutes. Alternative energy carriers in the transport sector are not explicitly modeled in any of the three CGE models, although implicit efficiency improvements in the transport sector are considered.

### Description of the model framework

**Fig. 1:**
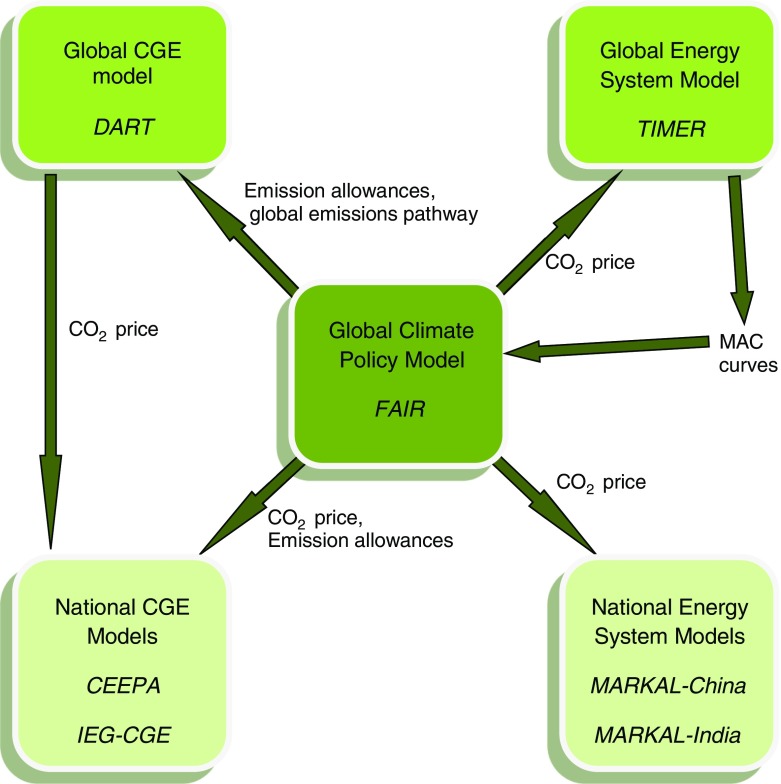
The figure shows a schematic overview of how the seven models are soft-linked in the applied model framework, and how outputs from some of the models are used as input to other models

In order to exploit the advantages of all seven models and compare the results from the different models, central features have been harmonized among the models. The models are also linked in the sense that the outputs from some models are used as input to other models. In the model framework, the FAIR model provides a bridge function, see Fig. 1.

The harmonization and linking between the models can be summarized as follows:

1. All models are harmonized to a common baseline scenario.
2. FAIR calculates the CO_2_-equivalent emissions^3^ pathway, a globally uniform carbon price and regional emission allowances based on the energy related CO_2_ part of the pathway and an effort sharing approach. Climate policy cost is also determined by FAIR.
3. DART determines the globally uniform carbon price and climate policy cost based on the global energy related CO_2_ pathway and the regional emission allowances from FAIR.
4. The national CGE models use the emission allowance from FAIR and the carbon price from DART to determine changes to the energy system and climate policy cost.
5. The national MARKAL models use the emission allowances and carbon price from FAIR to determine changes to energy system and climate policy cost.
6. TIMER uses the emission allowances from FAIR to determine changes to the energy system.

The reason for letting the national CGE models using CO_2_ prices from DART and the national MARKAL model using CO_2_ prices from FAIR (based on MAC-curves from TIMER) is that the models in each respective model class (CGE models vs Energy System models) have many common features. The CGE models also have a similar theoretical underpinning. As will be seen in the results (Fig. 3) letting the different models using different prices will not have any major impact on the result since the prices are very similar in DART and FAIR up to 2045. However, the CGE models (MARKAL models) would render too little (much) abatement in 2050 if they were using the FAIR (DART) prices in 2050.

## Basic modeling and scenario assumptions

### Baseline assumptions and model harmonization

The models are harmonized with respect to discount rate, population growth, Gross Domestic Product (GDP) growth and fossil fuel prices. The discount rate is set at 5 % as in the Global Energy Assessment (GEA 2012). Table 2 summarizes the key baseline assumptions for population and GDP. The population projection is in line with the medium variant of United Nations World Population Prospects (UNDESA 2011), with the global population projected to increase to about 9.1 billion people in 2050. The GDP growth rates are based on the reference scenario of the OECD Environmental Outlook, with the global economy projected to grow with a factor of about 4 (OECD 2012). Finally, developments in international fossil fuel prices towards 2035 are taken from the current policy scenario of the World Energy Outlook 2010 (IEA 2010). Prices are kept constant after 2035.

**Table 2:** The table presents the assumptions on population and GDP per capita that are harmonized among the models and used in the baseline scenarios

		World	India	China
Population (million people)	2010	6927	1214	1388
	2020	7691	1367	1467
	2050	9154	1614	1454
GDP per Capita (MER^a^, USD_2005_/yr)	2010	7268	965	3278
	2020	9375	1975	7186
	2050	19836	9944	22841

^a^Market exchange rate

### Global emission pathway

**Fig. 2:**
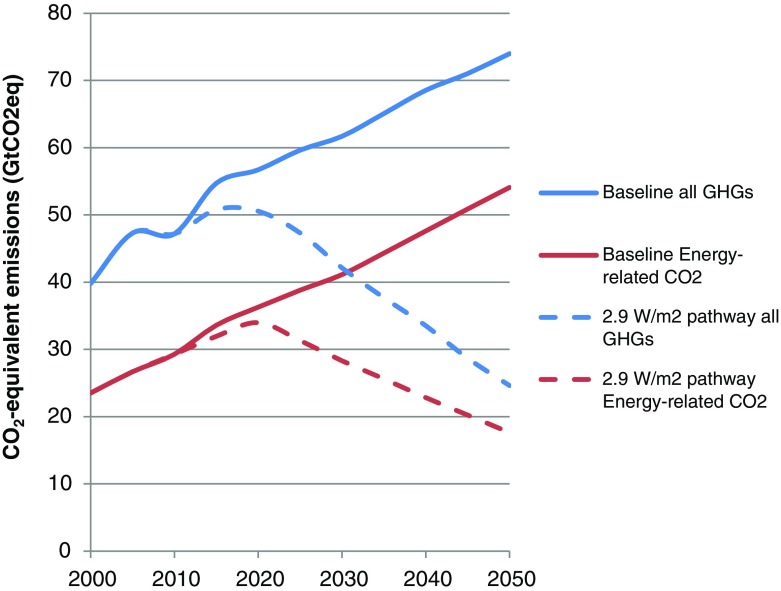
The figure shows, based on results from global climate policy model FAIR, global CO_2_-equivalent emissions (all greenhouse gases as included in the Kyoto protocol under UNFCCC) and energy-related CO_2_ emissions, for the baseline scenario and the 2.9 W/m^2^ pathway

**Fig. 3:**
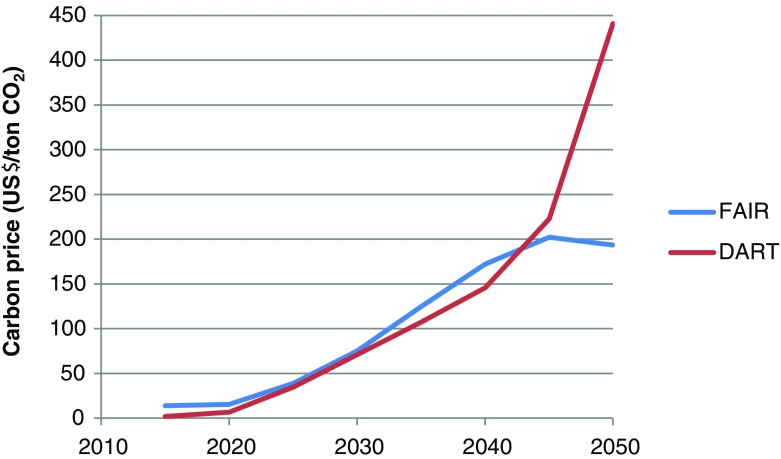
The figure shows carbon prices (in 2005 US$ value) compatible with the global emissions pathway from the global climate policy model FAIR and the CGE model DART to achieve the transition from the baseline emissions to the 2.9 W/m^2^ pathway

We constructed a global emission pathway that aims for a total radiative forcing of 2.9 W/m^2^ in 2100. This forcing level results, according to Meinshausen et al. (2006), in at least 50 % chance to stay within 2 °C temperature increase by 2100. The pathway implements the conditional, more ambitious emission pledges for 2020 presented in the Copenhagen Accord to the UNFCCC (Den Elzen et al. 2011).^4^ Between 2020 and 2025 global emissions gradually decline, while between 2025 and 2050 a constant reduction rate is assumed. For details see Lucas et al. (2013). Only the energy-related CO_2_ emissions from the CO_2_-equivalent pathway are used (see Fig. 2). Figure 3 presents the global CO_2_ price required in FAIR and DART to reach the pathway.

### Effort-sharing approach

An effort sharing approach is used to determine which part of the required global emission reductions is allocated to China and India, and which to other world regions. Here, we apply the so-called common-but-differentiated convergence (CDC) approach, a simple allocation scheme that takes into account common but differentiated responsibilities (Höhne et al. 2006). It assumes that per capita emission allowances of all countries converge, but that developing countries start their convergence trajectory only after reaching a certain threshold, e.g. per capita emissions or income. A similar differentiated convergence approach is discussed by He et al. (2009).

Important parameters for the CDC approach are the long-term per capita emission targets convergence level and the threshold that requires countries to enter the regime and start converging. Here, instead of a threshold, we define different country groupings based on 2009 Gross National Income (GNI) per capita (World Bank 2011), including developed countries, Advanced Developing Countries (ADC) and Other Developing Countries (ODC). Countries that according to their GNI per capita are high and upper middle income countries, and that are not already classified as developed countries, are classified as ADCs, and countries that according to their GNI per capita are low and lower middle income countries are classified as ODCs. After implementing their more ambitious Copenhagen pledges in 2020 (Den Elzen et al. 2011), the developed countries and the ADCs start converging instantly, China and India start in 2025 and 2030, respectively, and the remaining ODCs start in 2035.^5^ Between 2020 and the start of convergence countries follow their baseline trend.^6^ Developed countries converge in 2040, while all other countries take 30 years for convergence. All countries converge to a target level of 1.7 tCO_2_/capita, consistent with the global CO_2_ emissions pathway.^7^

## CO_2_ emissions, emission allowances and global carbon taxes

### Global results

The global greenhouse gas emissions, including all greenhouse gases and land use related emissions included in the Kyoto Protocol under the UNFCCC, and the corresponding energy related CO_2_ emissions generated by FAIR, are shown in Fig. 2. Without any mitigation policies, global greenhouse gas emissions and energy related CO_2_ emissions continue to increase towards 2050. The dotted lines in Fig. 2 represent the 2.9 W/m^2^ stabilization emissions pathway described in Section 3.2. While global greenhouse gas emissions peak before 2020, energy-related CO_2_ emission peak slightly later as reductions of non-CO_2_ emissions are more cost-effective in the short-term than reductions of energy-related CO_2_ emissions (Lucas et al. 2007). After peaking, emissions decrease gradually to 37 % below 1990 levels for all greenhouse gases and 17 % for the energy-related CO_2_ emissions, respectively, by 2050.

In DART and FAIR the transition from the baseline emissions to the 2.9 W/m^2^ pathway is achieved via a uniform carbon price on CO_2_ emissions (see Fig. 3). These prices are very similar up to 2045, beyond that the price in DART rises further, as mitigation options in DART are limited after certain abatement levels, while FAIR allows for more radical technology changes that become especially available in the long run due to technological change.

### Results for China

**Fig. 4:**
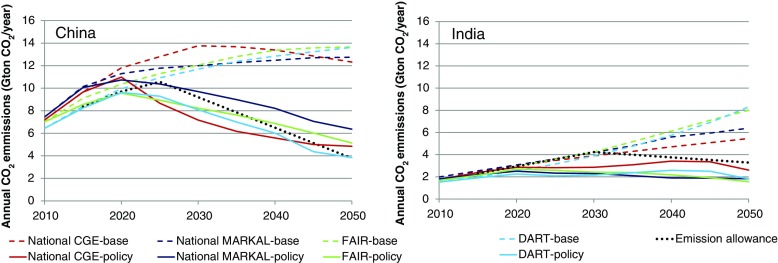
The figure shows energy-related CO_2_ baseline emissions, emission allowances in the climate policy scenario and emissions after trade in the climate policy scenario, for China (left panel) and India (right panel)

In the baseline scenario (without any international climate policies) CO_2_ emissions for China continue to increase in all models (Fig. 4 left panel). After 2030, a decrease in the growth rate can be observed even leading to a small decrease in absolute emissions in the CEEPA model. Since emissions were not harmonized among the models, there is a spread already in 2010.^8^ Interestingly, national models show considerably higher emissions in 2030 compared to the global models. This implies that meeting the emission pledges for 2020 presented in the Copenhagen Accord to the UNFCCC is much more challenging under these assumptions than in the global models. It should also be noted that China MARKAL considers some planned climate policies in the baseline scenario—such as the renewable energy development goal for the year 2020, the reduction of 40−45 % carbon intensity during 2005 to 2020. The other models also consider decoupling between energy demand and GDP, but do not consider explicit policies. The inclusion of these policies in the baseline in China MARKAL is one cause for the relatively low baseline emissions in that particular model towards the end of the time horizon. Furthermore, the final emissions according to the CDC regime—taking into account international emission trading—linger for most models and before 2035 slightly below the emission allowances, implying relative small revenues from international emissions trading. Only China MARKAL generates emissions (after emissions trading) under the CDC regime that are higher than the emission allowances for the whole time period, implying that, under our cost-optimal calculations, China is a net buyer of credits on the international carbon market. For the other three models China changes from being a seller to a buyer beyond 2035.

### Results for India

In the baseline scenario CO_2_ emissions in India continue to increase over the coming decades in all models (Fig. 4 right panel). The 2020 Copenhagen Accord pledge is almost identical or even slightly higher than baseline emissions in the different models. It should also be noted that MARKAL-India does consider some planned climate policies in the baseline scenario. The other models also do consider decoupling between energy demand and GDP, but do not consider explicit policies. IEG-CGE stands out here with the highest decoupling assumptions. The final emissions according to the CDC regime—taking into account international emission trading—remain considerably below the emission allowances in all models for the whole period between 2020 and 2050. This implies that India is a net seller of credits on the international carbon market.

## Energy system change and climate policy costs

### Changes in fuel mix

#### China

**Fig. 5:**
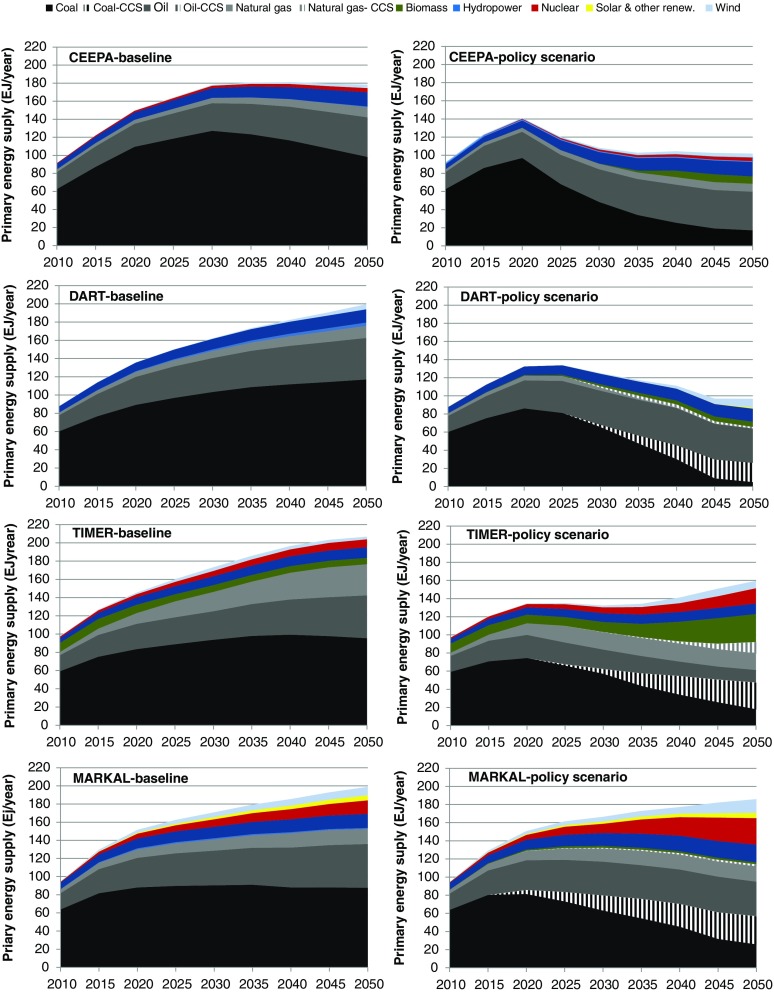
The figure shows the results on primary energy supply in China for the baseline scenario and the climate policy scenario from the different models

Figure 5 presents the Chinese primary energy supply generated by the models in the baseline and the climate policy scenario.^9^ Currently, the energy system is dominated by coal followed by oil. Other fuels such as natural gas and biomass play a less important role. The primary energy supply grows rapidly between 2010 and 2020, with 43–56 exajoules (EJ) in the baseline scenario. Between 2020 and 2050 primary energy supply grows on average at a lower annual rate, with an additional 20–56 EJ in the baseline scenario. Notable is that CEEPA shows a peak in primary energy supply by 2030 in the baseline, while the other models show continued growth. The peak in CEEPA is caused by a decline in the supply of domestic fossil fuels due to resource scarcity. This, together with the imperfect substitution between domestic and imported fuels (due to the Armington assumption), implies that domestic energy price increases and energy demand decreases.

In all models, coal remains the most important fuel in the baseline scenario; in 2050 it still contributes with about 50 % of the primary energy supply. Oil remains the next most important fuel up to 2050 in all models. Finally, natural gas consumption is projected to grow rapidly in all models, especially in TIMER.

In the climate policy scenario the energy supply in the models grows with 34–55 EJ between 2010 and 2020. Beyond 2020 the models indicate only a weak growth or even a decline in energy supply. Actually, a reduction of energy use stands out as a key mitigation option, especially in the CGE models (Fig. 5; see also Fig. 7 for a decomposition analysis of abatement activities). In CEEPA one reason for the reduction in energy demand under climate policy is that economic activity declines, while it increases in DART. Changes in economic activity are not considered in China MARKAL and TIMER (see Section 5.2.1). Other important abatement options are CCS (except for CEEPA) and increased use of biomass (primarily in TIMER) and nuclear energy (China MARKAL and TIMER).

A large difference across models is the degree to which technologies with low or zero CO_2_ emissions are deployed. The energy system models show higher shares of technologies with low or zero CO_2_ emissions than the CGE models, especially in the policy scenarios. In the energy system models, high carbon prices imply that the system starts investing mainly in technologies with low or zero CO_2_ emissions. It also means that less energy efficiency improvements are required to achieve the same level of emission reduction as compared to the CGE models (see Section 5.2). In addition, reduced fossil fuel demand seems to lead to a more rapid fall in international fossil fuel prices in DART than in the energy system models, leading to a negative feedback for the expansion of low carbon technologies.

#### India

**Fig. 6:**
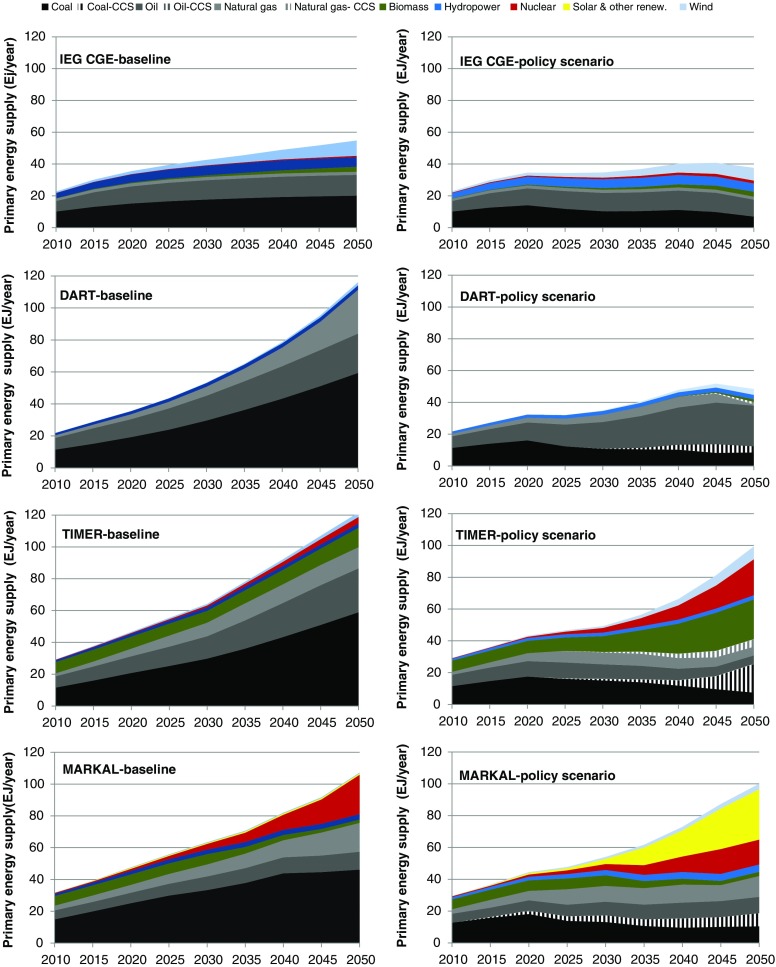
The figure shows results on primary energy supply in India for the baseline scenario and the climate policy scenario from the different models

The primary energy supply scenarios for India diverge in the different models (Fig. 6). Quite a large range of different energy demand levels are projected already for 2020: the lowest demand amount to 20 EJ in IEG-CGE while the highest amount to 50 EJ in MARKAL-India. However, one reason is that IEG-CGE does not include traditional biofuels, but more importantly, IEG-CGE shows a very high decoupling between energy and economic growth.

Similar as for China, it is projected that coal remains the most important fuel in the baseline scenario, followed by oil. In DART and TIMER, natural gas increases most rapidly. While natural gas also increases fast in MARKAL-India, it is outrun by nuclear power by 2050. This is attributed to the positive policy outlook towards building nuclear power capacity in the country, which is explicitly taken into account in MARKAL-India. This is not accounted for in IEG-CGE, DART and TIMER.

Again, the CGE models project a much larger role for reduction in energy consumption as an abatement option compared to the energy system models. Other important abatement options are CCS (all models except IEG-CGE), increased use of biomass (primarily in TIMER) and other renewable energy resources (mainly being different forms of solar energy in MARKAL-India; particularly PV). Similar to the results for China, the abatement in the energy system models depends to a stronger degree on biomass and other renewable energy sources than in the CGE models.

### Decomposition of abatement

To visualize key differences in abatement strategies across the models we undertake a decomposition analysis using the Kaya identity (Kaya 1990):

1 $$ {E}_{C{O}_2}(t)= GDP(t)\cdot ei(t)\cdot ci(t) $$

Where ${E}_{C{O}_2}$ is annual CO_2_ emissions, *GDP* the annual Gross Domestic Product, *ei* annual average energy intensity (i.e., unit primary energy per unit GDP) and *ci* annual average carbon intensity (i.e., unit CO_2_ emissions per unit primary energy). Based on additive decomposition techniques we analyze the contribution of *GDP*, *ei* and *ci* changes to total cumulative emissions reductions (Hoekstra and van den Bergh 2003).

#### China

**Fig. 7:**
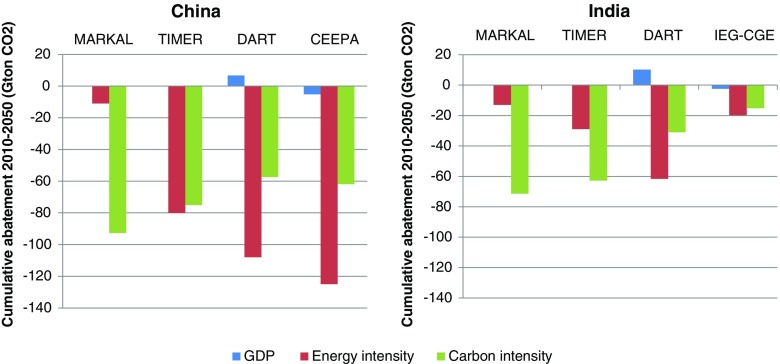
The figure shows the results of a decomposition of the different factors that contributes to the total 2010–2050 cumulative abatement in China (left panel) and India (right panel)

There are large differences in cumulative abatement and in how abatement occurs across the different models (Fig. 7 left panel). The total level of abatement in China is smaller in the energy-systems models compared to the CGE models (see Figs. 5 and 7 left panel).

One striking difference between the models is how much of the abatement is related to a decline in energy intensity versus carbon intensity. A reduction in energy intensity is the main abatement approach in TIMER, DART and CEEPA, while a large share of emission reductions in China MARKAL comes from a reduction in carbon intensity. The overall large decline in energy intensity in three of the four models indicates that efficiency improvements and structural changes within the economy are central for abatement in China. Yet, the decline in energy intensity does not only reflect end use efficiency improvements but rather total system efficiency improvements. Since there is a loss of conversion efficiency when using CCS, one observes smaller energy intensity reductions when CCS is an important technology. Hence, it is possible that the energy intensity contribution to emissions reductions in the models where CCS expands significantly underestimate actual energy end use efficiency improvements. In addition, in China MARKAL, energy conservation and efficiency improvements are considered in the baseline scenario leaving a smaller room for further efficiency improvements in the climate policy scenarios compared to other models which do not consider this in the baseline. Finally, since by construction China MARKAL is a technology focused model it does not consider the option for changes in energy services demand changes when relative prices change. However, this is taken into account in the other three models. All these aspects contribute to the lower contribution from energy intensity reduction in China MARKAL. Therefore, the use of renewable energy sources, nuclear power and CCS is considerably more important than energy efficiency measures for reducing emissions. These results are in line with the fact that renewable energy sources, nuclear power and CCS (and thus reductions in the carbon intensity) play a more important role in energy system models than in CGE models in general, and in particular in China MARKAL as seen in Fig. 5.

In the two CGE models GDP is affected by climate policies. CEEPA shows a loss in GDP in the climate policy scenarios as compared to the baseline scenario and for this reason the reduction in GDP contributes to further emission reduction. DART shows an increase in GDP as a result of climate policy, partly due to emissions trading, and partly due to a decline in fossil fuel prices. This contributes to increasing the emissions. In general, the contribution of GDP is small compared to the contributions of reductions in energy and carbon intensity.

#### India

Similar to the models results for China, abatement in India occurs differently in the different models (Fig. 7 right panel). The total level of abatement is smallest in IEG-CGE model. The main reasons are that the baseline emissions in this model are considerably lower than in the other three models,^10^ and that reducing emissions in this model is relatively costly.

Also, similar to the case for China, both CGE models (DART and IEG-CGE) mainly abate through a decrease in energy intensity (see Fig. 7). This fact indicates, again, that efficiency improvements and structural changes within the economy are central for abatement in these models. On the other hand, MARKAL-India obtains only a small reduction in emissions from decreased energy intensity. For TIMER, decreased energy intensity is important for abatement but not as important as a reduction in carbon intensity.^11^ A reduction in carbon intensity can be achieved via the use of CCS and renewable energy sources, and a switch from carbon intensive coal to less carbon intensive natural gas. In MARKAL-India virtually all abatement occurs through decreased carbon intensity. The reasons why MARKAL-India is showing only a small reduction in energy intensity in comparison to the other three models are identical to those for China MARKAL as discussed in Section 5.2.1.

In the two CGE models GDP is again affected by climate policies. GDP decreases in IEG-CGE due to climate policies and for this reason the reduction in GDP contributes to abatement. For DART the increases in GDP that follows from climate policies contribute to increasing emissions. As in the case of China the overall contribution of GDP to total cumulative abatement is relatively small.

### Direct and macro-economic costs of climate policy

The cost of climate policy is measured as abatement cost relative to baseline GDP levels in the energy system models (including FAIR) and as welfare changes (Hicks equivalent variation) relative to the baseline for the CGE models. The estimates for economic impacts are therefore not directly comparable between the two model classes. Furthermore, since the models include different technologies, sectors and energy sources it can be expected that abatement costs differ. Energy systems models focus on the competition between different technologies for meeting the demand for goods and services and derive cost estimates from detailed descriptions of the energy systems. In contrast, CGE models focus on the economy as a whole and include the interactions between the various sectors. They do not focus on direct costs, but on changes in economic production and consumption levels or welfare, which better captures overall structural changes and economy wide effects.

**Fig. 8:**
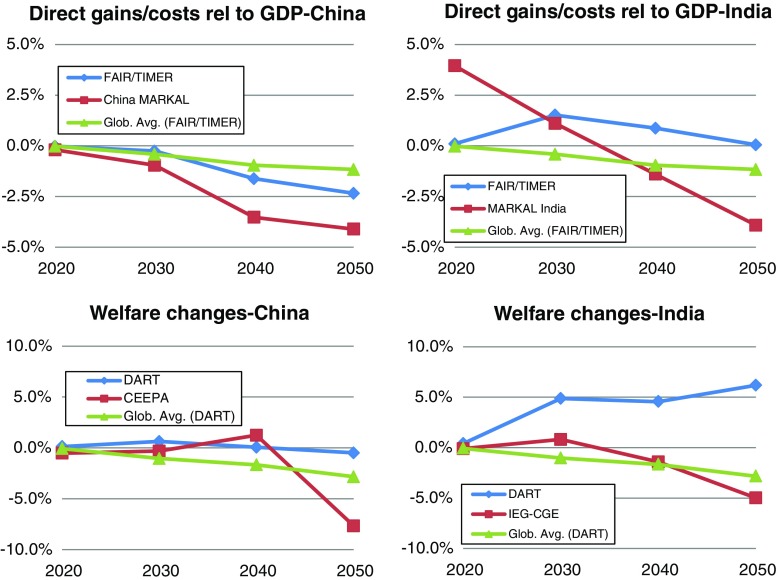
The figure shows the economic impacts of climate policy in China (left) and India (right) estimated by the different models. For FAIR and MARKAL gains or costs are reported as abatement cost relative to GDP (top), while for the CGE models welfare changes (Hicks equivalent variation) are reported (bottom)

The economic impacts of the climate policy scenario for China and India are depicted in Fig. 8. The figures also show the global average effects from FAIR and DART to put regional effects into perspective (for the economic burden of India and China relative to the global average (Hof et al. 2009; Van Ruijven et al. 2012b)).

#### China

In general, costs are increasing over time although there are large differences between the models. While the CGE models show moderate costs for a longer period, in the case of DART for the whole model period, costs increase to 2.5 or even 5 % relative to GDP in the energy system models by the end of the time horizon.

One explanation for modest cost estimate in DART is that in DART the repercussions on the international fuel market are relatively large. The world (as a whole) consumes less fossil fuels in the climate policy scenario as compared to the baseline scenario, so that the (global) fossil fuel price declines. China, an importer of fossil fuels, can profit from this, while energy exporting countries such as Russia lose export revenue. In CEEPA, this effect is not present (assumption of a small open economy with world prices fixed between the two scenarios). Also, neither FAIR nor China MARKAL capture this effect. Further, China is a net seller of credits up to 2050 in DART, while in CEEPA and FAIR, China is a seller until 2020, but becomes a net buyer afterwards (see Fig. 4). In China MARKAL, China is a net buyer of credits over the whole time period considered.

#### India

As expected, the climate policy scenario also affects India differently in the different models (Fig. 8 right). The global models, DART and FAIR, show an economic gain from international climate policies throughout the simulation period. The key reason is that the Indian emissions calculated by the models are substantially lower than the allocated emission allowances, see Fig. 4. As a consequence, India can, in the models, sell allowances on the international allowance market and generate revenues that are large enough to offset increased investment costs in energy efficient technologies and technologies with low or zero CO_2_ emissions. This is different from the model results for China, where the possibility to sell on the international market is much smaller (see Fig. 4). In addition, the Indian economy is smaller than the Chinese and for this reason an equal net export of carbon allowances in absolute terms has a larger impact on India in relative terms. FAIR shows a small benefit in 2020, a somewhat larger gain in 2030 and 2040, and a close to zero gain in 2050. The latter is caused by a reduction of exported allowances.

For the DART results it is again important that international fuels prices decline in the policy scenario compared to the baseline scenario causing benefits to net importers of fossil fuels such as India. This effect is again non-existing in FAIR, MARKAL-India and IEG-CGE. IEG-CGE shows a loss in welfare that grows over time, due to an increase in carbon prices. This is in stark contrast to the results found in DART. In IEG-CGE capital inflows (from selling allowances) lead to an appreciation of the Indian currency which lowers international competitiveness.^12^ This is modeled differently in DART and not at all considered in MARKAL-India and FAIR.

The result for MARKAL-India is also different. One cause is the inter-temporal optimization methodology. The perfect foresight assumption implies that the knowledge of future high CO_2_ prices causes investments and national fuel prices to decline early on in the model leading to an initial gain from climate policies. Later, costly investments in abatement technologies are needed and the benefit of climate policies found at earlier decades turns to a loss.

## Sensitivity analysis

Model results are sensitive to a broad range of assumptions. Here, we discuss the economic implications of an alternative GDP growth path, the timing of global emission reductions and two alternative effort sharing approaches. Global studies have in the past underestimated the economic growth in the emerging economies in Asia, particularly in China (Van Ruijven et al. 2012b). To address its impact, the models are run with a higher GDP growth scenario for China and India, while the rest of the world still follows our reference growth rate.^13^ With respect to the timing of global emission reduction, in our climate policy case countries implement their high Copenhagen Accord pledge in 2020, after which global emissions gradually decrease. In the existing literature, most studies have used cost optimal pathways, with global 2020 reductions generally being larger, while the mid- and long-term reductions can be slightly lower compared to our climate policy case (Den Elzen et al. 2011). Therefore, for comparison, we also run our models with a cost optimal pathway, resulting in global early action (Van Vliet et al. 2012). See also Lucas et al. (2013) for a discussion of energy system implications for the different assumptions on abatement timing. Finally, as national costs are highly dependent on how the global emission reductions are shared among countries, we assess the impacts of two alternative effort sharing approaches: a global uniform carbon tax approach and a delayed participation CDC approach. In the delayed participation CDC approach, China and India start converging 5 years later than in the base case.^14^ Table 3 shows the economic impacts of these alternative assumptions per region and model, using the 2010–2050 Net Present Value (NPV) of welfare impacts for the CGE models and direct abatement cost, including emissions trading, relative to GDP for the energy systems models.

**Table 3:** The table shows economic implications of alternative assumptions on economic growth, timing of global emission reductions and the effort-sharing approach, measured as 2010–2050 NPV. Positive numbers represent net gains and negative numbers net costs

		Reference case	Higher GDP growth	Global early action	Global uniform carbon tax	Delayed participation CDC
China	DART	0.2 %	−0.2 %	−0.2 %	−0.4 %	1.3 %
	CEEPA	−0.4 %	−2.4 %	−3.0 %	−9.7 %	3.6 %
	FAIR	−0.7 %	−1.0 %	−0.6 %	−0.7 %	−0.3 %
	China-MARKAL	−1.7 %	−2.9 %	−1.2 %	−1.0 %	−1.1 %
India	DART	4.0 %	3.9 %	3.0 %	−0.2 %	5.7 %
	IEG-CGE	−1.1 %	−1.6 %	−1.7 %	−2.0 %	0.0 %
	FAIR	0.7 %	0.7 %	0.1 %	−1.1 %	1.5 %
	MARKAL-India	1.7 %	−2.4 %	−1.0 %	−0.2 %	2.5 %

The economic impacts are generally larger for the two alternative effort sharing approaches, especially the global uniform carbon tax, compared to the alternative assumptions for economic growth and the global emission pathway. Higher economic growth and a global uniform carbon tax increase total climate policy costs for China and India, while delayed participation CDC results in lower climate policy costs for both countries. The impacts of global early action, i.e., higher global 2020 abatement, differ per country and model.

While the baseline emissions increase much more under the higher economic growth scenario, the emission allowances remain moreover the same, thus resulting in higher climate policy costs, especially for China. Since India remains a net seller of credits it continues to benefit from higher carbon prices. In sum, these two contradicting effects lead to only a small impact in both DART and FAIR, while in MARKAL-India, gains from emissions trading cannot offset the large increase in mitigation costs.

Global early action has a mixed impact on climate policy costs in the two countries and differs across models. For China, climate policy costs decrease in the energy system models and increase in the CGE models. The differences in results can at least in part be explained by the more detailed description of the capital stock turn-over in energy systems models. A later adoption of reduction targets implies a larger built up of fossil fuel based technology without CCS. Assuming that technologies are only replaced after their normal lifetime, the expected decreased demand growth in China implies that there are limits to the potential to reduce emissions, as there will be little demand for new facilities (see also Van Ruijven et al. 2012a). Hence, a less rapid emission reduction rate would be beneficial for Chinas in these models. In India, climate policy costs increase in all models, i.e., benefits either drop or costs increase. The increased costs due to a higher reduction objective in 2020 are not fully compensated through higher gains from selling allowances at higher carbon prices.

In both countries and all models delayed participation CDC results in reduced climate policy costs, while a global uniform carbon tax increases climate policy costs. This result is driven by the capital inflow from emissions trading, which is especially large for delayed participation CDC. Only China MARKAL shows decreasing climate policy costs under a global uniform carbon tax. Here, China is a net buyer of emissions credits in the whole 2010–2050 period in this model, while in all other models China becomes a net buyer only beyond 2035 (see Fig. 4).

## Discussion

In the prevailing literature, estimates on energy system and cost impacts of different climate regimes are often not directly comparable and differences in result are not always easy to explain (Van Ruijven et al. 2012b). We find in our analysis that models with a similar structure (CGE vs. energy system) lead to comparable results. Thus, differences in model results can be explained in part by the use of a CGE or an energy system model.^15^

In our analysis not all CGE models include technologies with low or zero CO_2_ emissions to the same extent as the energy system models (see Table 1) and thus react differently to climate policies. As a consequence the energy system models have more options for meeting the energy demand than CGE models and more abatement takes place via carbon intensity reductions, i.e., through changes in the energy supply mix (see Figs. 8 and 9). In the CGE models abatement primarily takes place via energy intensity reductions since these models offer more options for reducing energy demand and/or changing the structural composition of the production in the economy leading to production of less energy intensive goods.

Concerning cost estimates, CGE models take into account different kinds of repercussions on markets that the energy system models do not consider. Still the estimates on the cost of climate polices are comparable across the models. An important difference between the national CGE models and the global CGE model is that the global model takes into account repercussion on international fossil fuel markets. This has an important impact on the cost estimates, and results in lower overall costs for climate policies in India and China. Furthermore, impacts on the exchange rate following capital transfers from emission trading have a key impact on the effect climate policies has on welfare measures in the Indian CGE model. For both mechanisms see also Weitzel et al. (2012).

Finally, the models used in this paper are intentionally representing a strong simplification of the real world. This is important so as to make an interpretation of the results possible and since modeling of social-technical systems are utterly complicated. The key outcome from modelling exercises such as this one is not the exact numbers generated by the models, but rather the insights obtained. The key decision criteria used here is cost-effectiveness, i.e., social goals (such as climate targets) should be met at the lowest possible cost given various assumptions. Given this modeling approach the results presented in this paper represent future scenarios that are internally consistent in each model given the assumption at hand. Public opinion on technologies such as nuclear power and CCS may constrain or even inhibit large-scale expansion of such technologies. In our analysis we have intentionally left such issues aside and leave the implications of such issues to the user of the model results.

## Conclusions

This paper presents an overview of an international modeling comparison project, which focuses on how achieving the 2 °C target could affect economic and energy systems development in China and India. The analysis concludes that independent of models structure significant reductions are required in both China and India, implying huge changes in their energy systems.

In the main climate policy case (the common-but-differentiated convergence effort sharing approach), Indian emission allowances are allowed to grow more than the Chinese emission allowances, due to the per capita convergence rule and the higher population growth in India. In 2010, China’s CO_2_ emissions are almost three times higher than the Indian emissions, while in the baseline and policy scenarios in 2050 the CO_2_ emissions in China are about twice those in India. Demand for new capacity in India remains high towards 2050, while in China this demand levels off after 2030. As especially the energy-system models take account of the capital stock, this has a limiting effect on mitigation potential in China compared to India.

Clear differences and similarities with respect to the actual consequences for the energy system of climate policy can be observed, not only among the two countries, but also among the two model types. Energy efficiency improvements are more important in the CGE models, while improvements in the carbon intensity, primarily through expansion of CCS and renewable energy sources, are more important for the energy system models. With respect to the carbon intensity improvements, CCS is more important in China, while renewable energy sources (including biomass) are more important in India.

The negative economic impacts of international climate policy are generally larger in China than in India, and India can even gain economically. The reason for this is that India has a larger potential of selling reductions on the international carbon market generating revenues. In general, the model result show that China is a seller on the short term, but becomes a buyer on the long-term, while India is a seller over the whole 2010–2050 period. Dependent on the model, costs are also affected by decreasing global fossil fuel prices, currency appreciation resulting from a net capital inflow from international carbon trading and timing of emission reductions.

## Footnotes

1 In general as part of the Cancún Agreements, 42 developed countries (including the 27 EU Member States) have submitted quantified economy-wide emission reduction targets for 2020, and 48 developing countries submitted so-called nationally appropriate mitigation action plans reaching until 2020.

2 TIMER is part of the IMAGE integrated assessment model (Bouwman et al. 2006), but is here used as a stand-alone energy model.

3 All greenhouse gas emissions refer to all emissions relevant under the Kyoto Protocol (Annex A) including the land-use related CO_2_ emissions, i.e., the global warming potential-weighted sum of six Kyoto greenhouse gas emissions (CO_2_-equivalent emissions).

4 The emission resulting from pledges for the developing countries—including China and India—has been revised in this study due to a different baseline assumptions (OECD 2012), but keeping the same reduction below baseline from den Elzen et al. (2011).

5 According to the World Bank data, China and India are both classified as ODCs. As China’s per capita income in 2009 is almost reaching the upper-middle income threshold, it starts converging earlier than the other ODCs, but later than the ADCs. India also starts converging earlier than the other ODCs, but later than China.

6 Countries that made a 2020 pledge (including China and India) have similar reductions compared to their baseline emissions as in 2020 until they start converging.

7 Convergence to 1.7 tCO2/capita is consistent with the lower bound of IPCCs 2.5–3.0 W/m^2^ category of 80 –95 % reduction in 2050 for the group of aggregated developed countries (IPCC 2007).

8 This is because models have different calibration years (2004–2007) and are calibrated to different data sources.

9 When presenting results on primary energy supply from non-combustible and non-fossil energy (wind, hydro, other renewable and nuclear) we convert the electricity production from these sources by using a direct equivalent method, assuming a conversion efficiency of 35 % as in GEA (2012).

10 Although baseline emissions in IEG-CGE are very close to the projections of the National Council of Applied Economic Research until 2030 (Ministry of Environment and Forests and Government of India, 2009).

11 This is different from the situation in China where a slower increase in energy demand limits the transition from the existing fossil capital to low carbon technologies in TIMER– see also van Ruijven et al. (2012a).

12 Weitzel et al. (2012) analyze the effects of international fuel price and exchange rate repercussions on India in more detail. Further information (e.g. world market prices for fossil fuel) is passed on from DART to IEG-CGE.

13 For China, the national projection is based on Goldman Sachs (2010), IEA (2010), NBS Research Group (2011) and Li (2010). For India, we assume the high growth scenario of the Government of India (GOI 2006) extrapolated to 2050 by assuming declining growth rates after 2032 where the GOI study ends.

14 To stay within the global emission pathway, developed countries have to reduce more and converge to a target level of 0.6 tCO_2_/cap—instead of 1.7 tCO_2_/cap in the base case, while the developing countries still converge to a target level of 1.7 tCO_2_/cap. The global emission pathway is the same as in the reference climate policy case.

15 See for example Hourcade et al. (2006) and Van Vuuren et al. (2009) for further discussions on these types of models.
